# Supplementary material for: Apparent cooperativity between human CMV virions introduces errors in conventional methods of calculating multiplicity of infection
Source: eLife. 2026 Aug 5;14:RP108921. doi: 10.7554/eLife.108921 (PMC13441297; doi:10.7554/eLife.108921)
Supplement: Peer review history

# Editor report: eLife Assessment

**DOI:** 10.7554/eLife.108921.3.sa0

**Authors:** Frederik Graw

**Affiliations:** Friedrich-Alexander-University Erlangen-Nürnberg Germany

**Keywords:** Solid, Valuable

The authors combine experiments and mathematical modeling to determine how the infectivity of human cytomegalovirus scales with the viral concentration in the inoculum, i.e., considering the multiplicity of infection (MOI). They propose and test different model assumptions to explain a mechanism termed "apparent cooperativity" of virions based on an observed super-linear increase of the number of infected cells with increasing inocula. The authors present a **solid** study showing **valuable** findings for virologists and quantitative scientists working on the analysis and interpretation of viral infection dynamics for which quantitative knowledge of MOI is needed.

# Referee report: Reviewer #1 (Public review)

**DOI:** 10.7554/eLife.108921.3.sa1

Summary:

In this paper, the authors conduct both experiments and modeling of human cytomegalovirus (HCMV) infection in vitro to study how the infectivity of virus (measured by cell infection) scales with the viral concentration in the inoculum. A naïve thought would be that this is linear in the sense that doubling the virus concentration (and thus the total virus) in the inoculum would lead to double the fraction of infected cells. However, the authors show convincingly that this is not the case for HCMV, using multiple strains, two different target cells, and repeated experiments. In fact, they find that for some regimens (inoculum concentration) infected cells increase faster than the concentration of the inoculum, which they term "apparent cooperativity". The authors then provided possible explanations for this phenomenon and construct mathematical models and simulations to implement these explanations. They show that these ideas do help explain the cooperativity, but can't be conclusive as to what is the correct explanation. In any case, this advances our knowledge of the system and it is very important when quantitative experiments involving MOI are performed.

Strengths:

Careful experiments using state-of-the-art methodologies and advancing multiple competing models to explain the data.

Weaknesses:

Minor weaknesses in explaining the implementation of the model. However, some specific assumptions, which to this reviewer were unclear, could have substantial impact on the results. For example, whether cell infection is independent or not. This is expanded below.

In the revised version, the authors address almost all of these minor weaknesses, strengthening the paper and its reproducibility.

Suggestions to clarify the study:

In the revised version, the authors carefully consider these suggestions and provide further details, clarifications and even some new results. Regarding the question of how infection of a cell with one virus could lead to lower probability for a secondary infection, I think that it is possible that infected cells activate antiviral programs that lead, for example, to lower expression of surface receptors. This has been considered at least in hepatitis C virus infection. However, this is a minor point.

Overall, I think the revised version provides a sound study with relevant conclusions, and I thank the authors for their thoughtful consideration of my previous comments.

# Referee report: Reviewer #2 (Public review)

**DOI:** 10.7554/eLife.108921.3.sa2

In their article, Peterson et al. wanted to show to what extent the classical "single hit" model of virion infection, where always the same quantity of virion is required to infect a cell, does not match with empirical observations based on human cytomegalovirus in vitro infection model, and how this would have practical impacts in experimental protocols.

Strengths:

- The use of a very simple and robust experimental assay, where they infected cells with serially diluted virions and measured the proportion of infected cells with flow cytometry. This convincingly showed how the proportion of infected cells differed from a "single hit" model which they simulated using a simple mathematical model ("power-law model"), and better fitted a model where virions need to cooperate to infect cells.

- The use of different cell types and virus strains, which allows to draw some generalizations.

- The exploration of the mechanisms that could explain this apparent cooperation, using biologically plausible simulations.

- The practical consequences that this phenomenon has for lab virologists as well as modelers.

Weaknesses:

- The impossibility to discriminate between biological mechanisms is an important limitation of this study and calls for developing experimental designs able to further understand this question.

- The outcome of the virion clumping remains highly sensitive to the choice of the clumps size distribution, which is itself very complicated to estimate, especially at high dilution.

- The impossibility to directly fit the mathematical models to the data limit them to a qualitative discussion.

Overall, this work is very valuable as it raises the general question of how the estimate of infectivity can be biased if extrapolated from a single virus titer assay. The observation that HCMV virions often cooperate and that this cooperation varies between context seems robust. The putative biological explanations would require further exploration.

This topic is very well known in the case of segmented viruses and the semi-infectious particles, leading to the idea of studying "sociovirology", but to my knowledge this is the first time that it was explored for a non-segmented virus, and in the context of MOI estimation.

# Author comment: Author response

**DOI:** 10.7554/eLife.108921.3.sa3

**Authors:** Christopher Peterson, Joshua Miller, Brent Ryckman, Vitaly V Ganusov

**Affiliations:** University of Montana Missoula United States; University of Bristol Bristol United Kingdom; University of Montana Missoula United States; Texas Biomedical Research Institute San Antonio United States

The following is the authors’ response to the current reviews.

**Public Review:**

**Reviewer #1 (Public review):**

Suggestions to clarify the study:

In the revised version, the authors carefully consider these suggestions and provide further details, clarifications and even some new results. Regarding the question of how infection of a cell with one virus could lead to lower probability for a secondary infection, I think that it is possible that infected cells activate antiviral programs that lead, for example, to lower expression of surface receptors. This has been considered at least in hepatitis C virus infection. However, this is a minor point.

Yes, the possibility that infection of a cell by a virion would reduce chance of infection by another virion was allowed in our model. However, such as a process will not result in apparent cooperativity (n>1) in our model, and thus, is irrelevant to the issue of apparent cooperativity we identified.

**Reviewer #2 (Public review):**

In their article, Peterson et al. wanted to show to what extent the classical "single hit" model of virion infection, where always the same quantity of virion is required to infect a cell, does not match with empirical observations based on human cytomegalovirus in vitro infection model, and how this would have practical impacts in experimental protocols.

Strengths:

The use of a very simple and robust experimental assay, where they infected cells with serially diluted virions and measured the proportion of infected cells with flow cytometry. This convincingly showed how the proportion of infected cells differed from a "single hit" model which they simulated using a simple mathematical model ("power-law model"), and better fitted a model where virions need to cooperate to infect cells.

The use of different cell types and virus strains, which allows to draw some generalizations.

The exploration of the mechanisms that could explain this apparent cooperation, using biologically plausible simulations.

The practical consequences that this phenomenon has for lab virologists as well as modelers.

Thank you.

Weaknesses:

The impossibility to discriminate between biological mechanisms is an important limitation of this study and calls for developing experimental designs able to further understand this question.

The outcome of the virion clumping remains highly sensitive to the choice of the clumps size distribution, which is itself very complicated to estimate, especially at high dilution.

The impossibility to directly fit the mathematical models to the data limit them to a qualitative discussion.

Overall, this work is very valuable as it raises the general question of how the estimate of infectivity can be biased if extrapolated from a single virus titer assay. The observation that HCMV virions often cooperate and that this cooperation varies between context seems robust. The putative biological explanations would require further exploration.

This topic is very well known in the case of segmented viruses and the semi-infectious particles, leading to the idea of studying "sociovirology", but to my knowledge this is the first time that it was explored for a non-segmented virus, and in the context of MOI estimation.

Thank you. We would note, however, that inability to discriminate between alternative models is not a weakness per se. It shows that our work goes beyond a somewhat typical approach in mathematical modeling to offer a single explanation for a phenomenon in question (rather than focusing on discriminating between alternatives that is often hard to do).

**Recommendations for the authors:**

**Reviewer #2 (Recommendations for the authors):**

(1) I now understand better the graphical abstract. I think my eye was too much attracted by the increase in specific infectivity that you see for more than 1 genome/cell, which is not the point of your paper. I am wondering if you should not guide even more the reader, by pointing out that the fact that the initial decline in specific infectivity represents apparent cooperativity.

Let’s hope that the readers are smart enough to understand what to focus their eyes on. At the end, this is a graphical abstract that is not supposed to have too much text explaining where to look.

(2) For your one-inflated geometric distribution, I agree that the estimations would remain very hypothetical because you would have to make many assumptions, however I think a hurdle model where you would fit the P(clump size = 1)=f1 and P(clump size = i) following a one-truncated geometric distribution would be more appropriate because it would lead to a distribution closer to your PDF from figure S11C.

The issue is that our data are not in clump sizes but in diameter of the clump *D*. This is why we opted for using a mixture of continuous distributions, not a mixture of discrete distributions. We are sharing the DLS data, so others are welcome to do another try of fitting other types of distribution to the data.

(3) For the DLS data, I understand your choice to include all the datapoints, however I find the interpretation confusing: if I understand correctly, you consider that f1, the fraction of the smaller distribution, represents clumps of one virion. However, its median size is 10 times smaller than a virion. So, the number of clumps with one virion would be overestimated. I think it would be helpful for the reader to clarify this aspect, either in the results around lines 503-512, or in the discussion. Could it be that at higher dilution, what is represented by this smaller distribution would almost only be debris because the virions are so rare?

When fitting a mixture of two log-normal distributions f_1_ represents the proportion of clumps of larger size (as was described in the materials and methods). The actual estimated value of f_1_ is not highly relevant in calculating change in PDF of the distribution only for D>=d (230nm) as shown in Suppl Fig S11C. But we now realize that this variable f_1_ may be confused with a variable f_1_ used to denote the fraction of clumps with virion size=1 (in Fig 5C). We now mention that in the caption of Supp Fig S10.

(4) For the dashed diagonal lines of fig 2, what I don't understand is the choice of the intercept that seems a bit random. I was wondering if it would not be more helpful to make it so that the dashed line intersects the observation for 1 genome/cell, which could then be interpreted as a deviation from the "single hit" model extrapolated outside of 1 genome/cell?

The diagonal lines in Fig 2 are exactly the same in ALL panels, as are the x/y axes ranges; the slope of the line (equals to 1) allows visually to see when the regression (shown by think black lines) deviates from slope=1, i.e., indicates apparent cooperativity. We will keep the lines are they are. Thank you for the suggestion, though.

The following is the authors’ response to the original reviews.

**Reviewer #1 (Public review):**

Summary:

In this paper, the authors conduct both experiments and modeling of human cytomegalovirus (HCMV) infection in vitro to study how the infectivity of the virus (measured by cell infection) scales with the viral concentration in the inoculum. A naïve thought would be that this is linear in the sense that doubling the virus concentration (and thus the total virus) in the inoculum would lead to doubling the fraction of infected cells. However, the authors show convincingly that this is not the case for HCMV, using multiple strains, two different target cells, and repeated experiments. In fact, they find that for some regimens (inoculum concentration), infected cells increase faster than the concentration of the inoculum, which they term "apparent cooperativity". The authors then provided possible explanations for this phenomenon and constructed mathematical models and simulations to implement these explanations. They show that these ideas do help explain the cooperativity, but they can't be conclusive as to what the correct explanation is. In any case, this advances our knowledge of the system, and it is very important when quantitative experiments involving MOI are performed.

Strengths:

Careful experiments using state-of-the-art methodologies and advancing multiple competing models to explain the data.

Weaknesses:

There are minor weaknesses in explaining the implementation of the model. However, some specific assumptions, which to this reviewer were unclear, could have a substantial impact on the results. For example, whether cell infection is independent or not. This is expanded below.

Suggestions to clarify the study:

(1) Mathematically, it is clear what "increase linearly" or "increase faster than linearly" (e.g., line 94) means. However, it may be confusing for some readers to then look at plots such as in Figure 2, which appear linear (but on the log-log scale) and about which the authors also say (line 326) "data best matching the linear relationship on a log-log scale".

This is a good point. We included a clarification to indicate that linear on the log-log scale relationship does not imply linear relationship on the linear-linear scale. We wrote:

“Because most data did not exhibit a linear relationship between virion concentration and infection probability we fitted the models to subsets of data best matching a linear relationship on a log-log scale. Note that linear relationship on log-log scale may still be nonlinear (on linear-linear scale) when n!=1.”

(2) One of the main issues that is unclear to me is whether the authors assume that cell infection is independent of other cells. This could be a very important issue affecting their results, both when analyzing the experimental data and running the simulations. One possible outcome of infection could be the generation of innate mediators that could protect (alter the resistance) of nearby cells. I can imagine two opposite results of this: (i) one possibility is that resistance would lead to lower infection frequencies and this would result in apparent sub-linear infection (contrary to the observations); or (ii) inoculums with more virus lead to faster infection, which doesn't allow enough time for the "resistance" (innate effect) to spread (potentially leading to results similar to the observations, supra-linear infection).

In our models we assumed cells to be independent of each other (see also responses to other similar points). Because we measure infection in individual cells, assuming cells are independent is a reasonable first approximation. However, the reviewer makes an excellent point that there may be some between-cell signaling happening in the culture that “alerts” or “conditions” cells to change their “resistance”. It is also possible that at higher genome/cell numbers, exposure of cells to virions or virion debris may change the state of cells in the culture, and more cells become “susceptible” to infection. This is a good point that we now list in Limitations subsection of Discussion; it is a good hypothesis to test in our future experiments. We write:

“Accrued damage model is also consistent with the idea that at higher genome/cell values, the inoculum itself (including cell and/or virion debris) may impact overall susceptibility of all cells in the well, for example, making them more susceptible to infection. It may be expected, though, that exposing cells to debris would increase cell resistance to infection; this would result in n < 1 that we did not observe at small genomes/cell values.”

(3) Another unclear aspect of cell infection is whether each cell only has one chance to be infected or multiple chances, i.e., do the authors run the simulation once over all the cells or more times?

Each cell has only one chance to be infected. Algorithm 1 clearly states that; we will add an extra sentence in “Agent-based simulations” to indicate this point.

(4) On the other hand, the authors address the complementary issue of the virus acting independently or not, with their clumping model (which includes nice experimental measurements). However, it was unclear to me what the assumption of the simulation is in this case. In the case of infection by a clump of virus or "viral compensation", when infection is successful (the cell becomes infected), how many viruses "disappear" and what happens to the rest? For example, one of the viruses of the clump is removed by infection, but the others are free to participate in another clump, or they also disappear. The only thing I found about this is the caption of Figure S10, and it seems to indicate that only the infected virus is removed. However, a typical assumption, I think, is that viruses aggregate to improve infection, but then the whole aggregate participates in infection of a single cell, and those viruses in the clump can't participate in other infections. Viral cooperativity with higher inocula in this case would be, perhaps, the result of larger numbers of clumps for higher inocula. This seems in agreement with Figure S8, but was a little unclear in the interpretation provided.

This is a good point. We did not remove the clump if one of the virions in the clump manages to infect a cell, and indeed, this could be the reason why in some simulations we observe apparent cooperativity when modeling viral clumping. We have explored this in the revision and found that it does not really impact how infection rate scales with the genomes/cell (e.g., see Suppl Fig S8).

(5) In algorithm 1, how does P_i, as defined, relate to equation 1?

These are unrelated because eqn.(1) is a phenomenological model that links infection per cell to genomes per cell. P_i in algorithm 1 is “physics-inspired” potential barrier.

(6) In line 228, and several other places (e.g., caption of Table S2), the authors refer to the probability of a single genome infecting a cell p(1)=exp(-lambda), but shouldn't it be p(1)=1-exp(-lambda) according to equation 1?

Indeed, it was a typo, p(1)=1-exp(-lambda) per eqn 1. Thank you, it has been corrected in the revised paper.

(7) In line 304, the accrued damage hypothesis is defined, but it is stated as a triggering of an antiviral response; one would assume that exposure to a virion should increase the resistance to infection. Otherwise, the authors are saying that evolution has come up with intracellular viral resistance mechanisms that are detrimental to the cell. As I mentioned above, this could also be a mechanism for non-independent cell infection. For example, infected cells signal to neighboring cells to "become resistance" to infection. This would also provide a mechanism for saturation at high levels.

We do not know how exposure of a cell to one virion would change its “antiviral state”, i.e., to become more or less resistant to the next infection. If a cell becomes more resistant, there is no possibility to observe apparent cooperativity in infection of cells, so this hypothesis cannot explain our observations with n>1. Whether this mechanism plays a role in saturation of cell infection rate at lower than 1 value when genome/cell is large is unclear but is a possibility. We added this point to Discussion in revision (see our text above that includes this point).

(8) In Figure 3, and likely other places, t-tests are used for comparisons, but with only an n=5 (experiments). Many would prefer a non-parametric test.

We repeated the analyses in Fig 3 with Mann-Whitney test, results were the same, so we would like to keep results from the t-test in the paper.

**Reviewer #1 (Recommendations for the authors):**

(1) The strains of HCMV used have a fluorescent reporter "in place of the US11 gene". Can you provide a brief comment on whether and how this gene deletion affects HCMV replication?

US11 is a resident ER protein that is considered an "immune evasion factor". It promotes ERAD of MHC I and has no observable effect on replication of HCMV in cultured cells (Berger 2000 JVI, Wiertz 1996 Cell). We now add this information in Materials and methods section of the paper. We write:

“All BAC clones were modified to express green fluorescent protein (GFP) or the monomeric red fluorescent protein mCherry (mCherry) with En passant recombineering by replacing US11 with the eGFP or mCherry gene, respectively. US11 is a resident ER protein that is considered an “immune evasion factor”. It promotes ERAD of MHC I and has no observable effect on replication of HCMV in cultured cells [27, 28]. Infectious HCMV was recovered by electroporation of BAC-DNA into MRC5 cells which were then co-cultured with either HFFCs (TB and TR) or HFF-tet cells (ME).”

(2) I didn't understand what the section "Virus titer assays" refers to. When was this used? How or why is this different from the "Virus stock dilution and dose-response assay"? Also in this section, you refer to NHDF cells - can you provide more information about these? And how does a different type of cell affect the titer assay (here measured as infected cells), since this is one of the main points of your paper?

Apologies for the confusion. In Ryckman lab we routinely generate viral stock and titrate it using a specific cell type, Normal (or neonatal) Human Dermal Fibroblasts (NHDF). This way, the titer of the stock is consistent between experiments by different researchers in the lab. We then use standard 10-fold dilutions to define the number of infectious units per mL of the stock. We now name this subsection as “Quantification of viral stock infectivity using standard 10-fold dilutions”. After the stock was quantified, we then used that stock in our actual experiments with very small dilution factor df that allowed us to detect deviations of the rate of infection from single hit model.

(3) In many places, "powerlaw" is written. This is usually written as two words, "power law".

Because powerlaw comes together with “model”, we decided to use “power-law model”.

(4) Line 75: "have" instead of "has"?

(5) Line 84: "with" repeated.

Corrected, thank you.

(6) Line 116: This section "Cell lines" seems to describe three cell lines, "HFF cells and MRC5 cells" and then "EC" cells.

HFF cells are fibroblasts used in our main experiments and MRC5 cells are another type of fibroblasts. We used MRC5 cells in the first step of recovering infection HCMV from BAC DNA (electroporation). We clarified this in Materials and methods. We write:

“Cell lines. Human foreskin fibroblast cells (HFFCs or fibroblasts) and MRC5 cells (also fibroblasts) were cultured in Dulbecco’s modified Eagle’s medium (DMEM, Sigma) supplemented with 5% heat-inactivated fetal bovine serum (FBS, Rocky Mountain Biologicals, Missoula, MT, USA) and 5%Fetalgro (Rocky Mountain Biologicals, Missoula, MT, USA). We used MRC5 cells in the first step of recovering infection HCMV from BAC DNA (electroporation). For main experiments we used HFFCs as fibroblasts. Human retinal pigment epithelial cells (ECs or ARPE-19, American Type Culture Collection, Manassas, VA, USA) were cultured in a 1:1 mixture of DMEM and Ham’s F-12 medium (DMEM:F-12, Gibco) and supplemented with 10% FBS.”

(7) Line 188: Because the virus is double-stranded, do you have to divide the qPCR result by 2 to get genomes?

This is typically accounted for in our calculations of genome/cell.

(8) Line 200: Typically, one would write "500g" and not "500xg".

Corrected.

(9) Line 248: It would be clearer to write "cell type C different from cell type C2".

Here C and C_2 refer to actual numbers of cell in the titration/growth experiments, so it is comparing numbers, not cell types. We kept the relationship as it is.

(10) Definition of cell class: what is n in p_n, the total number of cells, or are these divided into n classes of resistance?

This part was incorrectly copied from an earlier version, both cell resistance and virion infectivity was sampled from normal distributions with different mean and variances (see Table 1). We corrected the text to reflect this.

(11) Line 272 to 273: Something seems to be missing, as the change of line doesn't make sense.

Thank you. Edited to improve readability. Now it reads

“Clumping hypothesis. In the basic model the number of virions a given cell is exposed to follows a Poisson distribution. However, it is well recognized that as virions are produced by infected cells, they may form clumps/aggregates; the number of virions per clump/aggregate may deviate from, for example, the Poisson distribution [33].”

(12) Line 283: How lambda is chosen is not indicated here, only later (line 424), but at this point, one can confuse it with lambda in equation 1. Is it the same? It also doesn't seem to be indicated in your Table 1.

The mean of the Poisson distribution in clump simulations lambda is not the same as lambda in eqn 1; we re-named the mean of Poisson distribution as lambda_c which is estimated by fitting a Poisson distribution to clump size distribution estimated from DLS experiments. Because it was dependent on the virus stock dilution, it is not listed in Table 1. However, we did perform additional simulations assuming lambda_c=2 (Suppl Fig S10).

(13) Equation 6: I understand that you mostly used kappa=0, but in equation 6, would it be positive or negative (if not zero)?

We probably expect kappa to be negative but we did not fully explore this extension of the model.

(14) Line 350: Instead of "infection rates" would "infection frequencies" be better?

We agree. Changed (also changed in the sentence above that line).

(15) Line 366: I found this sentence a bit awkward.

We edited it to the best of our ability to improve it.

“Importantly, for most HCMV strain-target cell combinations we estimated n>1 (Figure 2 and Supplemental Table S2). With n>1 increase in virion concentration (i.e., higher genomes/cell values) results in a higher than linear increase in the probability of a cell to be infected (eqn. (1)) indicating cooperation between virions at infecting cells. We call this phenomenon “apparent cooperativity”.

(16) Figure 2, panel L: I wonder if it would be better to include the panel with the name of the experiment, but no data. Currently, it takes a while to find what you are talking about in panel L (or at the very least, indicate the panel in the caption).

Changed

(17) Figure 2: When you say that experiments were done at least twice, are you referring to the GFP and mCherry versions of the experiment, or replicates within each of those fluorescent labels?

Replicates with each of those labels.

(18) Figure 3: What is the number on top of the black bars? I think it is the average of the paired fold change. Is this right? Why, in panel E, is it 1.32 when only one goes up?

Yes, fold change. Indeed, 1.32 was a typo, it is 0.70, thank you for noting.

(19) Line 408: delete the word "there".

Done. Thank you.

(20) Line 412: Instead of "The", it should be "Then".

Done. Thank you.

**Reviewer #2 (Public review):**

In their article, Peterson et al. wanted to show to what extent the classical "single hit" model of virion infection, where one virion is required to infect a cell, does not match empirical observations based on human cytomegalovirus in vitro infection model, and how this would have practical impacts in experimental protocols.

They first used a very simple experimental assay, where they infected cells with serially diluted virions and measured the proportion of infected cells with flow cytometry. From this, they could elegantly show how the proportion of infected cells differed from a "single hit" model, which they simulated using a simple mathematical model ("powerlaw model"), and better fit a model where virions need to cooperate to infect cells. They then explore which mechanism could explain this apparent cooperation:

(1) Stochasticity alone cannot explain the results, although I am unsure how generalizable the results are, because the mathematical model chosen cannot, by design, explain such observations only by stochasticity.

Our null model simulations are not just about stochasticity; they also include variability in virion infectivity and cell resistance to infection. We agree that simulations cannot truly prove that such variability cannot result in apparent cooperativity; however, we also provide a mathematical proof that increase in frequency of infected cells should be linear with virion concentration at small genome/cell numbers.

(2) Virion clumping seemed not to be enough either to generally explain such a pattern. For that, they first use a mathematical model showing that the apparent cooperation would be small. However, I am unsure how extreme the scenario of simulated virion clumping is. They then used dynamic light scattering to measure the distribution of the sizes of clumps. From these estimates, they show that virion clumps cannot reproduce the observed virion cooperation in serial dilution assays. However, the authors remain unprecise on how the uncertainty of these clumps' size distribution would impact the results, as most clumps have a size smaller than a single virion, leaving therefore a limited number of clumps truly containing virions.

As we stated in the paper, clumping may explain apparent cooperativity in simulations depending on how stock dilution impacts distribution of virions/clump. This could be explored further, however, better experimental measurements of virions/clump would be highly informative (but we do not have resources to do these experiments at present). Our point is that the degree of apparent cooperativity is dependent on the target cell used (n is smaller on epithelial cells than on fibroblasts) that is difficult to explain by clumping which is a virion property. Per comment by reviewer 1, we have done more analyses of the clumping model to investigate importance of clump removal per successful infection on the detected degree of apparent cooperativity. We found that it was not critical to our conclusions (Suppl Fig S8).

The two models remain unidentifiable from each other but could explain the apparent virion cooperativity: either due to an increase in susceptibility of the cell each time a virion tries to infect it, or due to viral compensation, where lesser fit viruses are able to infect cells in co-infection with a better fit virion. Unfortunately, the authors here do not attempt to fit their mathematical model to the experimental data but only show that theoretical models and experimental data generate similar patterns regarding virion apparent cooperation.

In the revision we now provide examples of our earlier simulations that “match” experimental data with a relatively high degree of apparent cooperativity (Supp Fig S9).

Finally, the authors show that this virions cooperation could make the relationship between the estimated multiplicity of infection and viruses/cell deviate from the 1:1 relationship. Consequently, the dilution of a virion stock would lead to an even stronger decrease in infectivity, as more diluted virions can cooperate less for infection.

Overall, this work is very valuable as it raises the general question of how the estimate of infectivity can be biased if extrapolated from a single virus titer assay. The observation that HCMV virions often cooperate and that this cooperation varies between contexts seems robust. The putative biological explanations would require further exploration.

This topic is very well known in the case of segmented viruses and the semi-infectious particles, leading to the idea of studying "sociovirology", but to my knowledge, this is the first time that it was explored for a nonsegmented virus, and in the context of MOI estimation.

Thank you.

**Reviewer #2 (Recommendations for the authors):**

Major comments:

Two aspects of the work would benefit from further thought:

(1) The simulation of virion clumps: in both cases (Poisson distribution or one-inflated geometric distribution), the proportion of clumps containing more than one virion will be small. For the Poisson distribution, as you fit the powerlaw model on the range of genomes/cell < ~ 3 genomes/cell (Figure 4B). I wonder to what extent this explains the sudden rise in infections/cells you observe above that limit. It would be interesting to plot the (cumulative) distribution of the clump sizes at different dilution levels to have a better idea.

The reviewer has a good eye, indeed, the relationship between infection frequency and genomes/cell is linear up to a point, and we believe the inflection point reflects the genomes/cell values when clumps contain more than 1 virion. Here is the results of simulations with distribution of virions/clump plotted:

Similarly, for the one-inflated geometric distribution, the proportion of clumps of size 1 is the sum of two events: f1, plus 1-f1 times the probability that the geometric distribution is zero, if I follow the methods on lines 287-294. I wonder if this is appropriate regarding the estimates made with the DLC. In particular, Figure 5C shows that the proportion of clumps of size 1 is more than ~ half of all the clumps, and does not seem to be the same distribution as the estimates made on Figure S9C. Maybe a hurdle model would be more appropriate?

This is a fair point. In our analyses we found that modeling clump size distribution is tricky and required various assumptions. The issue with the DLS data is that we do not really know the distribution of intact virions per clump so how to relate the size of the clump to the number of virions in a clump is wide-open; we explored several possibilities and found that the answer (whether clumping results in apparent cooperativity) depends on assumptions of how clumps are modelled (e.g., compare Fig 4B and Suppl. Fig S11). Hurdle model is not appropriate for clumps because by our definition of a clump, it must have at least 1 virion. Our key observation, however, is that the degree of apparent cooperativity depends on the target cell type – and thus should be independent of virion clumping (unless there is viral cooperativity in the clumps). Overall, we decided that exploring more clumping models would take extra effort, but it is unclear if it brings any benefits to our conclusions.

The analysis of the clump size distribution using dynamic light scattering, in Figure S8. If I interpret correctly, events with size < 230 nm should be excluded as they do not represent clumps of virions but rather media impurities or cell debris. Therefore, I don't understand the choice of fitting the whole set with a combination of two normal distributions, as even the larger normal distribution covers clumps < 230 nm. If the f1 indicated here is the one used in the methods line 287-294, this is then wrong because it does not represent the fraction of clumps of size 1, but rather debris.

We used two normal (on log-scale) distributions when quantifying clump distribution data (Supp Fig S10) to avoid sub-selection of the data; in this way, two distribution fit the whole dataset with excellent quality. An alternative approach would be to sub-select data with size >=230nm and fit a normal (or similar) distribution of the clumps; such an approach may generate biases and/or unreliable estimates at high dilutions due to small number of clumps with large size (e.g., see Supp Fig S10S-X). In our simulations to model clump distribution and infection (Fig 5) we attempted to simulate the estimated clump size distribution (Suppl Fig S11C) only approximately. Again, because in our measurements we don’t really know the number of virions per clump, efforts to model exactly clump size distribution, we believe, are not going to give full answers.

(2) Figure 4 and results lines 419-465: Why didn't you try to fit the different models to the data, instead of qualitatively comparing the estimate of n in the simulations with arbitrary parameters to the one for empirical data? Your models match the expectation of virion cooperation by design, so they are not more convincing for a virologist than logical non-quantitative reasoning. They would be of stronger evidence in my opinion if you could show how well they fit the data. You could then directly compare the different models' fits using goodness-of-fit metrics and decide whether one is better than another or if they all explain equally well the observations.

Well, we have 11 different relationships between infection rate and genome/cell, finding parameter combinations that would match all the data with at least 2 alternative models seems excessive at present but it is a good direction as we get extra funding to continue this work. It is also difficult to extensively search for the parameter values that would result in a perfect fit of the stochastic simulations to data since the methods of fitting agent-based models to data are not fully developed. However, following this suggestion we now show results of simulations for the two alternative models (accrued damage and viral compensation) that we believe do match experimental data somewhat (see new Suppl Fig S9).

Minor comments:

(1) Graphical abstract: This requires more context as it is too rough here to help me understand the general idea of the paper. Plus, why does specific infectivity first decrease with genome/cell?

We added few elements to the graphical abstract including the strain and target cell used. The decrease in specific infectivity at lower genome/cell is due to apparent cooperativity.

(2) Equation (7): It would be beneficial for the reader if the reasoning behind the likelihood computation were further described.

This is a relatively standard approach to model/estimate parameters of a binary outcome, e.g., see Wikipedia: https://en.wikipedia.org/wiki/Logistic_regression

(3) Line 352-357: could the drop in infectivity also be enhanced/explained by increased cell mortality? Did you gate on cell viability during FCM?

The infection rate was measured in live cells only, so increased cell mortality may be an explanation.

(4) Figure 2: I don't understand the dashed diagonal lines: what do they represent exactly? Especially, wouldn't the single-hit model depend on p(1), in which case it should vary by cell x virus?

As the caption to Figure 2 clearly states, diagonal dashed lines show the slope = 1 (i.e, single hit model), so one would be able compare how far the data and/or model fit line deviate from 1. The note for p(1) in panel A is to illustrate how p(1) is calculated; obviously it varies by the strain-cell combination as is indicated in Suppl. Tab S2.

(5) Fig3G: Is it not surprising to find a positive relationship between p(1) and n? I would have intuitively expected that the stricter the environment is, the more cooperation you observe. But maybe these viruses did not evolve in this context, and therefore, this relationship is different from what you expect from an evolutionary optimum.

Well, we simply don’t know. The relationship simply suggests that there is connection between infectivity of a single virion and the degree of apparent cooperativity. We are not certain what is the context in which these viruses have evolved.

(6) Flow cytometry assay: could it be possible that cells infected by more virions generate more fluorescent proteins and are therefore less likely to be false negatives? Maybe you could compare the fluorescence intensity distribution among infected cells in the context of low MOI vs high MOI?

This is an interesting point. From presented flow cytometry plots (e.g., Suppl Fig S3), the MFI for infected cells does not seem to depend on the dilution (or genome/cell).

(7) Figure S9B: I did not understand this figure. Are the axes labels correct? How is it possible to have less than 1 virion/well?

The y axis shows a scaled number calculated from integrating estimated clump size distribution, we assume 1 “scaled” virion/well at highest virion/cell values. With scaling, yes, it is possible to have less than 1 virion/well.

**Reviewer #3 (Public review):**

Summary:

The authors dilute fluorescent HCMV stocks in small steps (df ≈ 1.3-1.5) across 23 points, quantify infections by flow cytometry at 3 dpi, and fit a power-law model to estimate a cooperativity parameter n (n > 1 indicates apparent cooperativity). They compare fibroblasts vs epithelial cells and multiple strains/reporters, and explore alternative mechanisms (clumping, accrued damage, viral compensation) via analytical modeling and stochastic simulations. They discuss implications for titer/MOI estimation and suggest a method for detecting "apparent cooperativity," noting that for viruses showing this behavior, MOI estimation may be biased.

Strengths:

(1) High-resolution titration & rigor: The small-step dilution design (23 serial dilutions; tailored df) improves dose-response resolution beyond conventional 10× series.

(2) Clear quantitative signal: Multiple strain-cell pairs show n > 1, with appropriate model fitting and visualization of the linear regime on log-log axes.

(3) Mechanistic exploration: Side-by-side modeling of clumping vs accrued damage vs compensation frames testable hypotheses for cooperativity.

Thank you.

Weaknesses:

(1) Secondary infection control: The authors argue that 3 dpi largely avoids progeny-mediated secondary infection; this claim should be strengthened (e.g., entry inhibitors/control infections) or add sensitivity checks showing results are robust to a small secondary-infection contribution.

This is an important point. We do believe that the current knowledge about HCMV virion production time – it takes 3-4 days to make virions per multiple papers (see Fig 7 in Vonka and Benyesh-Melnick JB 1966; Fig 3B in Stanton et al JCI 2010; and Fig 1A in Li et al. PNAS 2015) – is sufficient to justify our experimental design but we do agree that an additional control to block novel infections with would be useful. We had previously performed experiments with a HCMV TB-gL-KO that cannot make infectious virions (but the stock virions can be made from complemented target cells). We will investigate if our titration experiments with this virus strain have sufficient resolution to detect apparent cooperativity. However, at present we do not have the resources to perform novel experiments.

(2) Discriminating mechanisms: At present, simulations cannot distinguish between accrued damage and viral compensation. The authors should propose or add a decisive experiment (e.g., dual-color coinfection to quantify true coinfection rates versus "priming" without coinfection; timed sequential inocula) and outline expected signatures for each mechanism.

Excellent suggestion. Because infection of a cell is a result of the joint viral infectivity and cell resistance, it may be hard to discriminate between these alternatives unless we specify them as particular molecular mechanisms. But we tried our and listed potential future experiments in the revised version of the paper. Specifically, we write:

“Second, while we have proposed alternative mechanisms that may result in apparent cooperativity, at present we could not discriminate between these alternatives, in part, because the models lacked specifics – e.g., if virions interacting with a cell reduce its resistance to infection, what does it mean exactly [12]? If virions in a collection augment their infectivity (which may be expected for segmented viruses), how does that viral compensation actually work? Designing experiments that would discriminate between these alternatives would require focusing on a specific mechanism. For example, it may be that that the initiation of gene expression is difficult but is more efficient when there are more virions bringing in more tegument transactivators like pp72/ppUL35 [59]. Alternatively, it may be that there is a bona fide resistance mechanism at play here (e.g. “interferon”) that is antagonized by a viral tegument protein (like TRS1/IRS1 that acts against PKR and 2’5’OAS) [60]. Accrued damage model is also consistent with the idea that at higher genome/cell values, the inoculum itself (including cell and/or virion debris) may impact overall susceptibility of all cells in the well, for example, making them more susceptible to infection. It may be expected, though, that exposing cells to debris would increase cell resistance to infection; this would result in n < 1 that we did not observe at small genomes/cell values. Addressing these hypotheses is an area of future research that will require funding.”

(3) Decline at high genomes/cell: Several datasets show a downturn at high input. Hypotheses should be provided (cytotoxicity, receptor depletion, and measurement ceiling) and any supportive controls.

Another good point. We do not have a good explanation, but we do not believe this is because of saturation of available target cells. It seemed to only happen (or was most pronounced) with the ME stocks, which are typically lower in titer and so the higher MOI were nearly undiluted stock. It may be the effect of the conditioned medium. Or perhaps there are non-infectious particles like dense bodies (enveloped particles that lack a capsid and genome) and non-infectious, enveloped particles (NIEPs) that compete for receptors or otherwise damage cells and these don’t get diluted out at the higher doses. We included the point about cell death in Discussion of the revised version of the paper. Specifically, we write:

“We also do not have a clear explanation of why infection frequency declines at high genomes/cell values for some strain-cell combinations (e.g., Figure 2A, C, D, I, J). Because we measured cell infection in live cells, increase in cell death at higher genomes/cell values may result in the decrease in the number of viable cells.”

(4) Include experimental data: In Figure 6, please include the experimentally measured titers (IU/mL), if available.

This is a model-simulated scenario, and as such, there is no measured titers.

(5) MOI guidance: The practical guidance is important; please add a short "best-practice box" (how to determine titer at multiple genomes/cell and cell densities; when single-hit assumptions fail) for end-users.

Good suggestion. We now include best-practice box using guidelines developed in Ryckman lab over the years in the revised version of the paper. This is how it reads:

“Match viral titration methods to the experiment as far as possible. This includes using the same dilution of the viral stock, the cell type, duration of inoculation, and readout of infection.

When possible, determine the degree of apparent cooperativity (“n”-value, eqn. (1)) for each virus strain/cell type pair being studied.

If n = 1 (no cooperativity), it is reasonable to calculate experimental MOI based on stock infectivity value determined from a convenient stock dilution.

If n > 1 or unknown, then stock infectivity should be determined at a dilution resulting in an MOI as close as possible to the desired experimental MOI. Alternatively, the inoculum size can be empirically determined to yield the desired number of infected cells. In these ways different virus/cell type pairs can be compared more fairly.

Box 1: Recommendations on titrating viral stocks and on performing experiments when comparing different viral strains.”

**Reviewer #3 (Recommendations for the authors):**

FROM PUBLIC REVIEWS (2) Discriminating mechanisms: At present, simulations cannot distinguish between accrued damage and viral compensation. The authors should propose or add a decisive experiment (e.g., dual-color coinfection to quantify true coinfection rates versus "priming" without coinfection; timed sequential inocula) and outline expected signatures for each mechanism.

This is a good point but to propose a good experiment we need to narrow down the “generic” mechanism to specific processes/genes. We put forward some ideas but clearly more work is needed here:

“Second, while we have proposed alternative mechanisms that may result in apparent cooperativity, at present we could not discriminate between these alternatives, in part, because the models lacked specifics – e.g., if virions interacting with a cell reduce its resistance to infection, what does it mean exactly [12]? If virions in a collection augment their infectivity (which may be expected for segmented viruses), how does that viral compensation actually work? Designing experiments that would discriminate between these alternatives would require focusing on a specific mechanism. For example, it may be that that the initiation of gene expression is just difficult but is more efficient when there are more virions bringing in more tegument transactivators like pp72/ppUL35 [59]. Alternatively, it may be that there is a bona fide resistance mechanism at play here (e.g. “interferon”) that is antagonized by a viral tegument protein (like TRS1/IRS1 that acts against PKR and 2’5’OAS) [60]. Accrued damage model is also consistent with the idea that at higher genome/cell, the inoculum itself (including cell and/or virion debris) may impact overall susceptibility of all cells in culture, for example, making them more susceptible to infection. It may be expected, though, that exposing cells to debris would increase cell resistance to infection; this would result in n < 1 that we did not observe at small genomes/cell values. Addressing these hypotheses is an area of future research that will require funding.”

(1) Methods transparency: Include raw spreadsheets or tables of dilution factors and per-well genome estimates used for Figure 1A; this will help reproducibility of the df = 1.3-1.5 pipeline.

Provided as supplemental xlsx file.

(2) Epithelial vs fibroblast contrast: Since n is lower on epithelial cells, expand on cell-intrinsic barriers that could dampen apparent cooperativity, and if this argues against simple clumping.

Indeed, this is our point that we raised in Discussion. Since ECs show lower n than fibroblasts, this observation argues against clumps. Going forward the contrast between cell types will be an approach to understand mechanism. One difference is entry pathways, the ECs involve endocytosis and endosome acidification whereas the fibroblasts do not. There are clearly different receptors involved also, although they are not clearly characterized. One recent report that might be relevant is Ohman 2024 PNAS that shows the gH/gL/UL128-131 complex (aka, "pentamer") is not just dispensable for entry into fibroblasts, but inhibitory. They suggest that the pentamer might bind to a receptor on fibroblasts that activates a pathways that acts against viral IE expression, It could be that in this situation, more virions are really helpful to overcome that block, whatever it is. We now update this point in Discussion.

(3) Visualization: In Figure 2, consider showing confidence bands for the fitted slope (n) within the colored fit window and reporting n {plus minus} SE in the panels.

Because we used custom scripts to fit models to data, showing bands of model predictions was a bit complex and would interfere with data points. But we now show 95% Cis for the estimated value *n* (that are listed in Suppl. Tab S2).

(4) Symbols: Define all symbols (e.g., V₀, n) on first use in the main text, not only in Methods.

Done.

(5) Plot axes check: Explain non-uniform axis labeling ("genomes/cell," "infections/cell").

This comment was unclear – which labels were not “uniform”? Genomes/cell indicate the expected number of genomes (or virions) that a cell is on average exposed to, infections/cell indicates the probability that a cell actually gets infected.

(6) Confidence interval for estimated parameters: Figure 3 A-C, please report estimated parameter intervals.

These are listed in Suppl. Tab S2. Putting Cis for all estimates would clutter the figure making it hard to tell which CIs are for which estimate. But we put the Cis for estimated parameter *n* in Figure 2.
